# Identification of a prognostic long noncoding RNA signature in lung squamous cell carcinoma: a population-based study with a mean follow-up of 3.5 years

**DOI:** 10.1186/s13690-021-00588-2

**Published:** 2021-04-28

**Authors:** Rongjiong Zheng, Mengdi Zheng, Mingming Wang, Feijie Lu, Meiling Hu

**Affiliations:** 1 Ningbo Urology and Nephrology Hospital, Ningbo Yinzhou No 2. Hospital, Ningbo, China; 2 Cixi People’s Hospital of Zhejiang Province, Zhejiang, China

**Keywords:** Lung squamous cell carcinoma, LncRNAs, Survival

## Abstract

**Background:**

Lung squamous cell carcinoma (LSCC) is a form of cancer that is associated with high rates of relapse, poor responsiveness to therapy, and a relatively poor prognosis. The relationship between long non-coding RNA (lncRNA) expression and LSCC patient prognosis remains to be established.

**Methods:**

In the present study, we discovered that lncRNAs were differentially expressed in LSCC tumor tissues relative to normal control tissues, and we explored the prognostic relevance of these lncRNA expression patterns using data from the Cancer Genome Atlas (TCGA).

**Results:**

These multidimensional data were analyzed in order to identify lncRNA signatures that were associated with LSCC patient survival outcomes. Kaplan-Meier survival curves revealed prognostic capabilities for three of these lncRNAs (LINC02555, APCDD1L-DT and OTX2-AS1). A Cox regression analysis revealed this three-lncRNA signature to be significantly associated with patient survival. Further GO and KEGG analyses revealed that the predicted target genes of these three lncRNAs were also potentially involved in cancer-associated pathways.

**Conclusions:**

Together these results thus indicate that this novel three-lncRNA signature can be used to predict LSCC patient prognosis.

**Supplementary Information:**

The online version contains supplementary material available at 10.1186/s13690-021-00588-2.

## Background

Lung cancer is a highly heterogeneous disease, with genetic, epigenetic, and environmental factors all acting to shape its development and progression. Lung cancer mortality rates are the highest of all forms of cancer, accounting for 25 and 30% of all cancer-associated deaths in the USA and China, respectively [1, 2]. In 2015 alone, 733,000 new cases of lung cancer were diagnosed in China (69% in males and 31% in females), while 218,527 new cases were diagnosed in the USA during this same period (52% in males and 48% in females). SEER data indicate that lung cancer patients exhibit a 5-year survival rate of just 18.1% [3]. Lung squamous cell carcinoma (LSCC) cases account for a significant fraction of overall lung cancer cases [4]. LSCC more often occurs in men, is related to the smoking of tobacco, and is often associated with high rates of relapse, poor responsiveness to therapeutic intervention, and a generally poor patient prognosis [5, 6]. While there have been many advances in the field of clinical oncology as a whole in recent years, rates of 5-year overall survival (OS) for LSCC patients still remain low. As such, it is vital that novel approaches be identified that can be used to predict the prognosis of LSCC patients so as to guide clinical decision making and treatment efforts in these individuals.

Long non-coding RNAs (lncRNAs) are RNA molecules > 200 nucleotides in length that lack coding potential [7]. Emerging evidence indicates that some lncRNAs do encode proteins and play roles in transcriptional, and epigenetic gene regulation, and cancer [8, 9]. These lncRNAs have been shown to frequently be dysregulated in cancer, with their altered expression patterns having a direct impact on tumor cell gene expression at the post-transcriptional and epigenetic levels, as well as on the proliferation, survival, invasion, and metastasis of these cells [10–12]. However, relatively few studies to date have specifically examined the relationship between lncRNA expression and LSCC patient prognosis. In the present study, we therefore explored patterns of differential lncRNA expression in LSCC tumor tissues and normal control tissue samples in an effort to assess the prognostic relevance of such lncRNA expression patterns. Through this approach we were able to develop a three lncRNA signature which was found to be significantly associated with the survival of LSCC patients.

## Materials and methods

### LSCC patient datasets

Level 3 expression and clinical data pertaining to 409 LSCC and 49 control samples were downloaded from The Cancer Genome Atlas (TCGA, https://tvga-data.nci.nih.gov/tcga/). Datasets and patient records were used in order to assess both patterns of lncRNA expression as well as clinocpathological and demographic variables including gender, age at time of diagnosis, and TNM staging (Table 1). The mean follow-up time was 3.5 years. The Ethics Committee of the Institutional Review Board of Ningbo Yinzhou Second Hospital and Cixi People’s Hospital in Zhejiang Province approved this study. Samples were included in the present analysis if they were from patients with an OS > 1 month for whom lncRNA differential expression data and information pertaining to clinical details and prognosis were available. The *language* package in R was used in order to interpret the lncRNA sequencing data, while the *limma* package was used when assessing differential lncRNA expression between LSCC and control samples, with differential expression being expressed based upon fold change (FC) values. Those lncRNAs with a log_2_|FC| > 1.0 and *p* < 0.05 were considered to be significantly differentially expressed.

**Table 1:** Baseline characteristics of all patient samples: a population-based study with a mean follow-up of 3.5 years

Variables	All samples (*n* = 458)	
	No.	%
Gender		
Female	122	26.6
Male	336	73.4
Age at diagnosis		
> 60	360	78.6
≤ 60	98	21.4
Stage		
I	224	48.9
II	150	32.8
III	77	16.8
IV	7	1.5
T stage		
T1	104	22.7
T2	269	58.7
T3	65	14.2
T4	20	4.4
Metastasis		
M0	376	82.1
M1	7	1.5
MX	75	16.4
Lymph node status		
N0	291	63.5
N1	119	26.0
N2	37	8.1
N3	5	1.1
NX	6	1.3

### Statistical analysis

The prognostic relevance of differentially expressed lncRNAs in LSCC was assessed using Kaplan-Meier curves and log-rank tests. We ultimately constructed a signature using a linear combination of the expression levels of these three lncRNAs and the estimated regression coefficients in the multivariable Cox regression analysis. A mathematical formula (Risk score = 0.0768*LINC02555 + 0.0917* APCDD1L-DT – 0.1176*OTX2-AS1) was developed to predict the risk score for each patient based on the multivariable Cox regression analysis. This three lncRNA signature-derived risk score was then used to stratify patients into high- and low-risk groups, using the median risk score in this cohort as a cutoff point for stratification purposes. Kaplan-Meier curves and log-rank tests were then used to compare survival outcomes between these high- and low-risk patients. In addition, receiver operating characteristic (ROC) analyses were used in order to compare the sensitivity and specificity of this three lncRNA risk score as a means of predicting patient survival outcomes. *P* < 0.05 was the significance threshold. R version 3.5.1 [13] was used for all statistical testing.

### Functional analysis

Correlating genes to the differentially expressed lncRNAs were obtained using the co-expression method. Pearson correlation coefficients between the expression profiles of the three prognostic lncRNAs and their protein-coding genes (PCGs) were calculated to determine their relationships. Those PCGs with a Pearson’s R > 0.40 and *p* < 0.05 were considered to be lncRNA-related. These putative lncRNA targets were then subjected to Gene Ontology (GO) and Kyoto Encyclopedia of Genes and Genomes (KEGG) functional enrichment analyses. Furthermore, these target genes were incorporated into a protein-protein interaction (PPI) network using the STRING database [14], with Cytoscape being used for network visualization [15]. Select protein pairs from this network with > 10 nodes were the outputs of this analysis.

## Results

### Patient characteristics

**Fig. 1:**
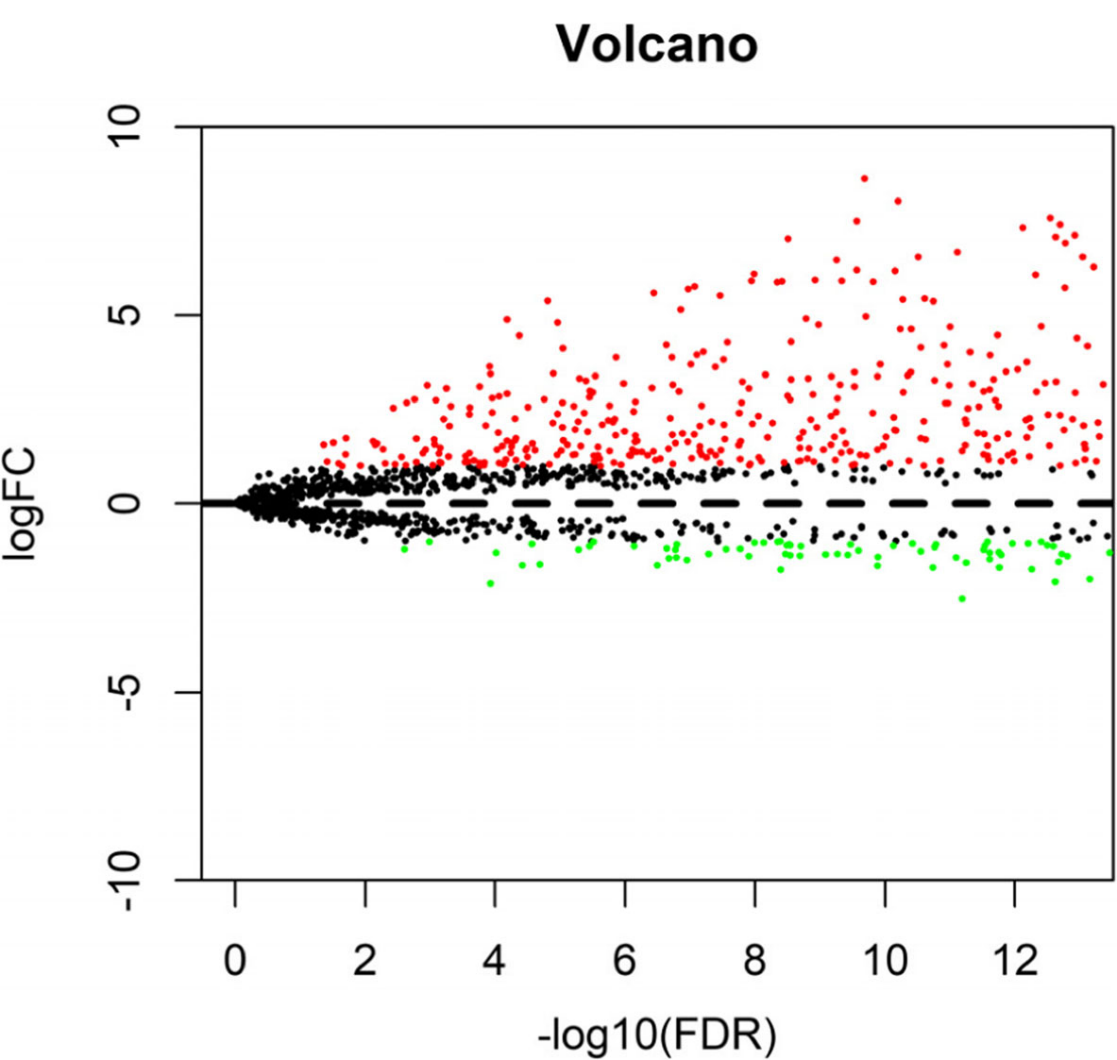
Volcano plot of differentially expressed lncRNAs. Red and green dots represent upregulated and downregulated lncRNAs, respectively: a population-based study with a mean follow-up of 3.5 years

The study investigated 458 patient samples. The mean follow-up time was 3.5 years and 200 patient samples were dead in this study. Table 1 lists detailed clinical characteristics, including gender, race, age at diagnosis, and disease stage. Of the enrolled patients, 26.6% were female, and 78.6% were older than 60 years. The most common tumor grades were I (48.9%) and II (32.8%). A total of 936 differentially expressed lncRNAs, including 687 upregulated and 249 downregulated lncRNAs, were identified between LSCC and normal tissues in Fig. 1.

### The relationship between lncRNA expression and LSCC patient OS

**Fig. 2:**
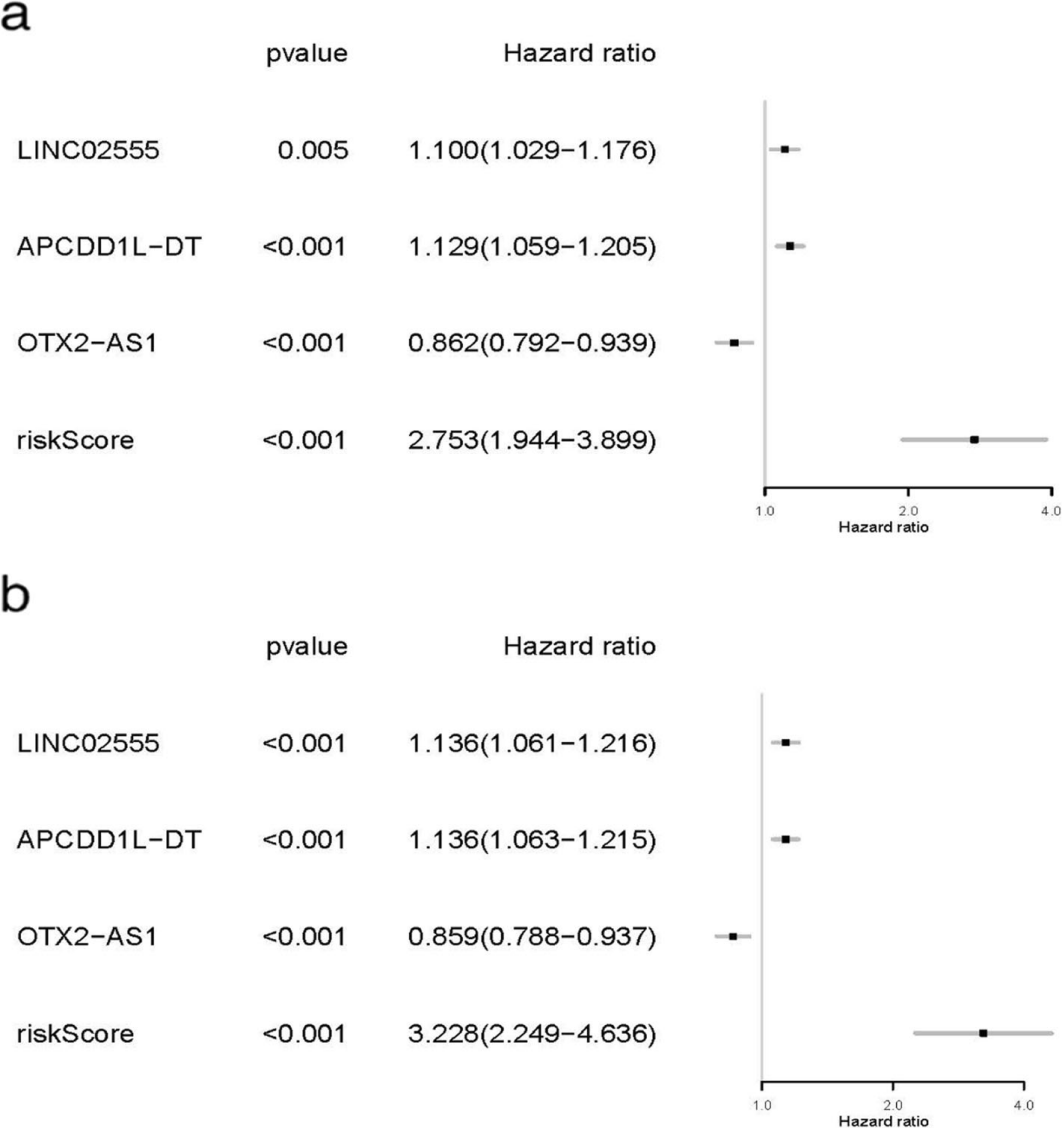
Forest plot for the association between three lncRNAs and risk value (**a**) Univariable Cox regression model (**b**) Multivariable Cox regression model: adjusted for age, gender and the stage: a population-based study with a mean follow-up of 3.5 years

**Fig. 3:**
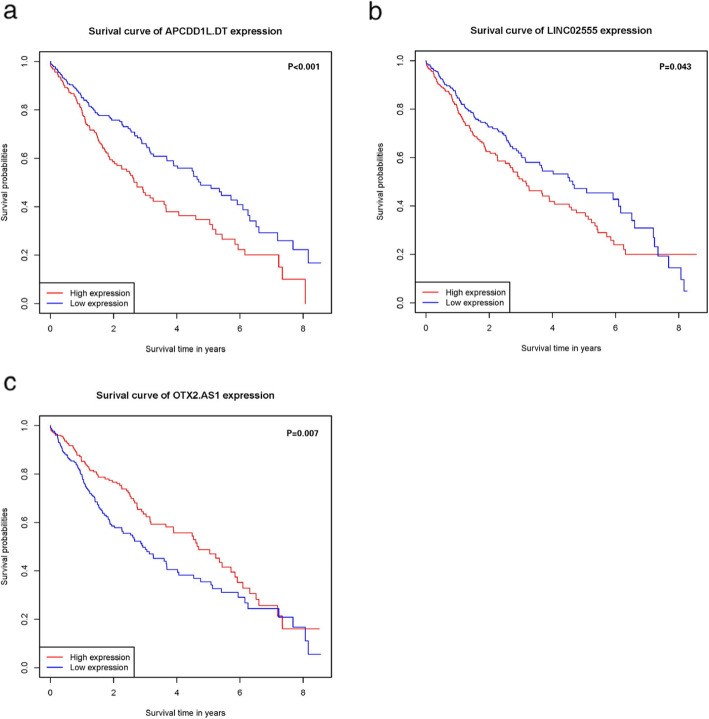
Kaplan-Meier method and log-rank test revealed that three lncRNAs were associated with OS in patients with LSCC. The patients were divided into low and high expression levels group according to the median value. **a** APCDD1L-DT **b** LINC02555 and (C) OTX2-AS1: a population-based study with a mean follow-up of 3.5 years

We began by using univariable and multivariable Cox proportional hazard regression models in order to identify those lncRNAs which were associated with LSCC patient prognosis. In total, we identified three candidate lncRNAs in these LSCC patients in univariable Cox model (*p* < 0.01; Fig. 2a). A multivariable model confirmed that the expression of the lncRNAs LINC02555 (HR = 1.136, *p* < 0.001), APCDD1L-DT (HR = 1.136, p < 0.001), and OTX2-AS1 (HR = 0.859, p < 0.001) were all independently associated with LSCC patient OS (Fig. 2b). Kaplan-Meier survival curves and log-rank tests were further used to examine the relationship between these lncRNAs and patient survival. We found that two of the tested lncRNAs (LINC02555 and APCDD1L-DT) were negatively associated with LSCC patient OS, whereas the lncRNA OTX2-AS1 was positively correlated with OS (Fig. 3).

### The prognostic utility of the three lncRNA signature

**Fig. 4:**
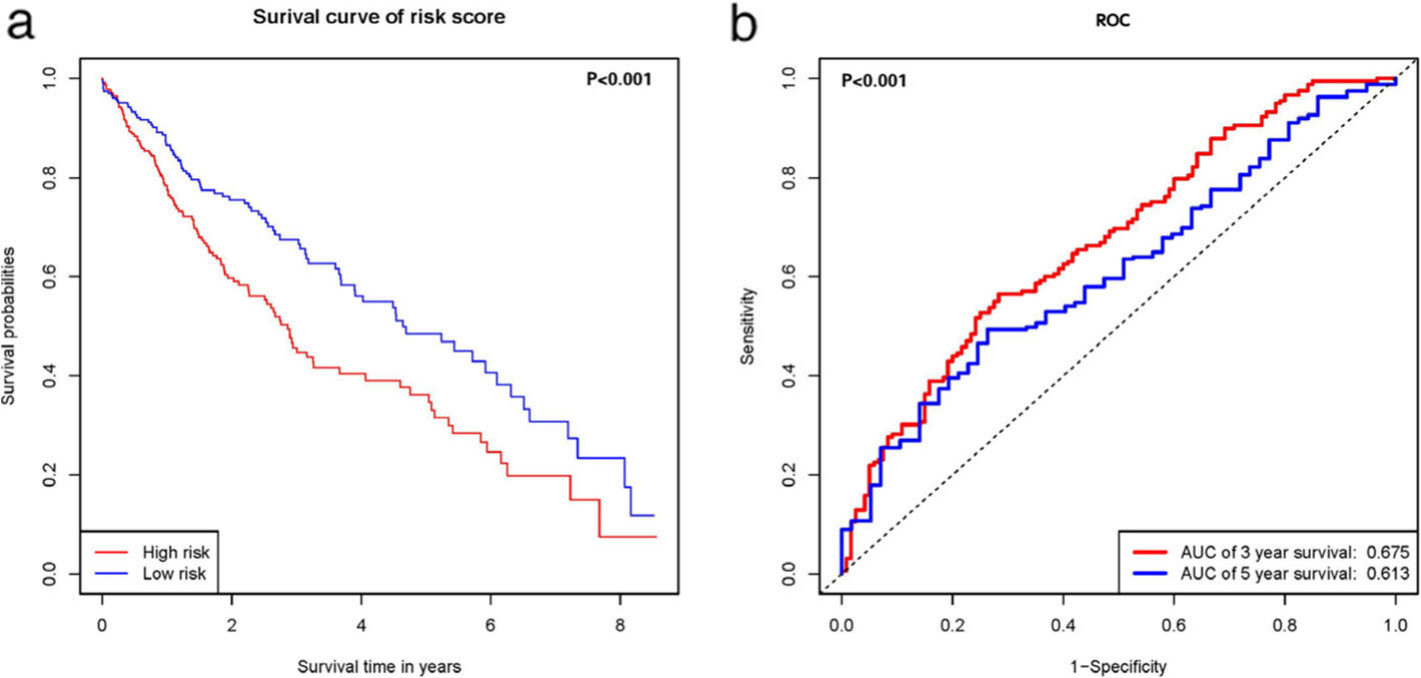
(**a**) Kaplan-Meier estimates of the survival outcomes for patients using the three-lncRNA signature, and (**b**) Receiver operating characteristic analysis of risk factors for survival prediction: a population-based study with a mean follow-up of 3.5 years

Using this three lncRNA signature, we were able to assign risk scores to patient samples, after which these samples were separated into high- and low-risk groups based upon the median risk score value. We then found that patients in the high-risk group had a significantly shorter OS than did patients in the low-risk group (*p* < 0.001) (Fig. 4a). We then used an ROC analysis in order to assess the prognostic utility of this three lncRNA signature. The AUC values for these curves as predictors of LSCC patient 3- and 5-year survival were 0.675 and 0.613, respectively, corresponding to an effective survival prediction (Fig. 4b). Patients in the high-risk group expressed higher levels of the lncRNAs LINC02555 and APCDD1L-DT on average relative to low risk patients, whereas low-risk patients expressed higher levels of OTX2-AS1 lncRNA.

### Functional enrichment analysis and PPI networks

**Fig. 5:**
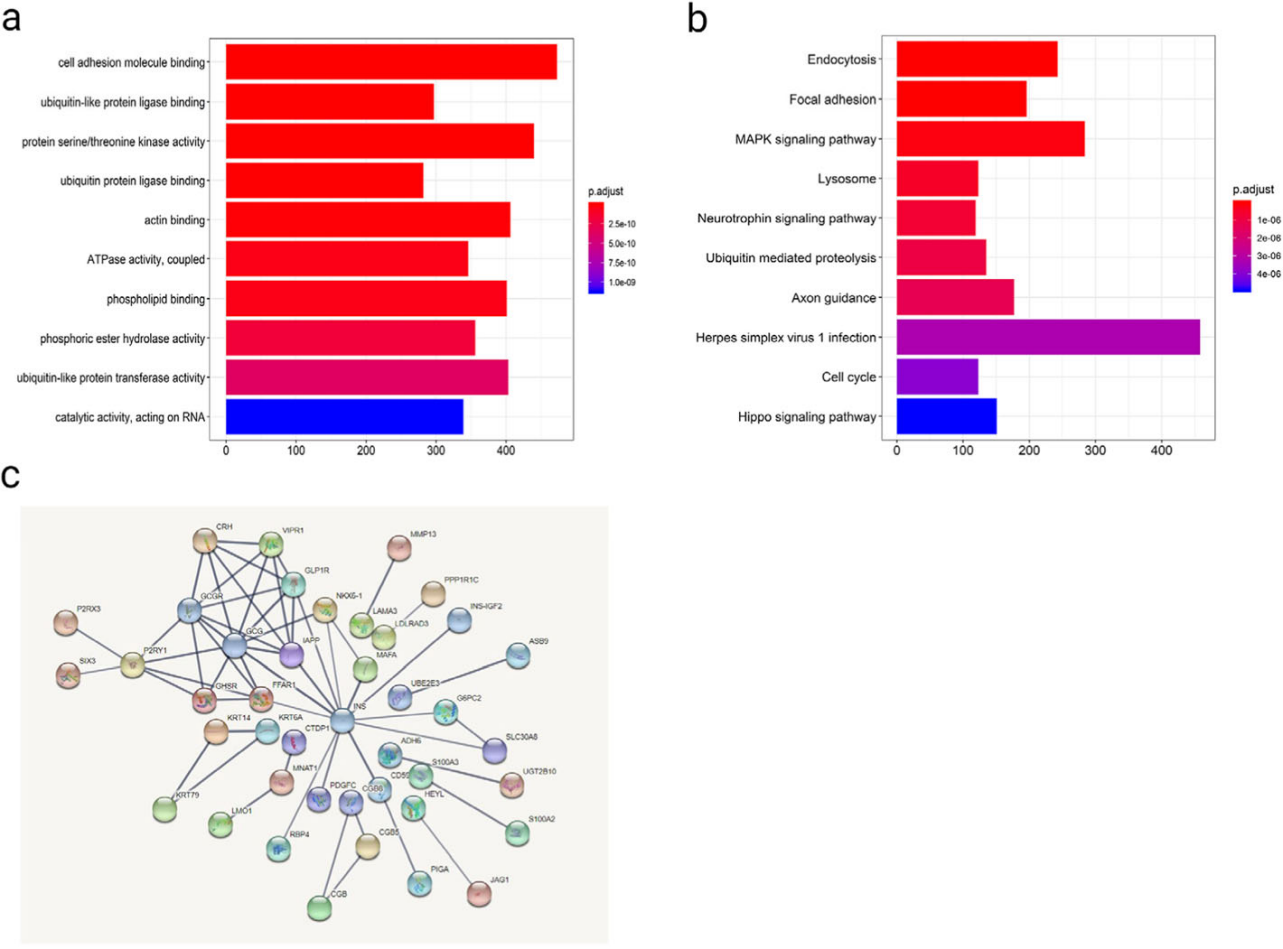
(**a**) The Gene Ontology and (**b**) Kyoto Encyclopedia of Genes and Genomes pathway enrichment analyses, and (**c**) PPI networks: a population-based study with a mean follow-up of 3.5 years

In order to identify potential targets for these three lncRNAs which were associated with LSCC patient prognosis, we conducted a co-expression analysis as detailed in the Materials and Methods section. We then performed GO and KEGG pathway analyses on these co-expressed genes in order to unravel their potential physiological roles (Fig. 5). These co-expressed genes were primarily enriched in genes associated with biological processes such cell adhesion molecule binding, ubiquitin-like protein ligase binding, protein serine/threonine kinase activity, ubiquitin protein ligase binding, actin binding, ATPase activity, phospholipid binding, and phosphoric ester hydrolase activity. These genes were additionally significantly enriched in KEGG pathways including endocytosis, focal adhesion, MAPK signaling, lysosomes, neurotrophin signaling, ubiquitin mediated proteolysis, axon guidance, herpes simplex virus 1 infection, and the cell cycle. Furthermore, PPI networks were also obtained using the STRING tool. As mentioned in Fig. 5c, INS, GCG, GCGR, GLP1R, IAPP, P2RY1, CRH and FFAR1 had the most connections with other members of the module and were thus the most noteworthy nodes in this network.

## Discussions

Lung cancer currently ranks as the deadliest form of cancer, and as such it is a primary focus for many cancer research efforts [16]. While LSCC patient prognosis has improved significantly in recent years owing to improvements in multidisciplinary treatment strategies, and chemotherapeutic/radiotherapeutic treatment regimens, LSCC recurrence rates remain high and as such this disease can impose a heavy burden upon patients, their families, and on medical institutions [17, 18]. Difficulties in accurately diagnosing LSCC and in predicting patient outcomes have led to low 5-year survival rates in affected patients [2]. As such, it is vital that novel biomarkers that can reliably predict LSCC patient outcomes be identified. It is similarly important that the molecular mechanisms governing the development and progression of LSCC be fully elucidated.

Many studies have clearly shown that the development of LSCC can be driven by interactions between genetic, transcriptomic, and proteomic factors [19, 20]. Changes in lncRNA expression patterns can also influence all stages of the oncogenic process, yet the prognostic relevance of these lncRNAs has not been sufficiently studied to date. As such, in the present study we examined lncRNA expression patterns in LSCC and were thus able to identify three lncRNAs that were significantly linked with LSCC patient OS. These three lncRNAs were then subjected to additional analyses aimed at identifying their putative target genes and potential biological roles through the use of pathway enrichment analyses. These results indicated that these three lncRNAs may play roles in regulating LSCC molecular pathogenesis, clinical progression, and patient prognosis, thus clearly demonstrating the prognostic relevance of lncRNA expression patterns in LSCC patients in a clinical setting.

Multiple studies [21, 22] have demonstrated that functional lncRNA expression can modulate oncognesis via altered regulation of gene expression and signaling within tumor cells. Indeed, certain lncRNAs are able to promote the development, progression, and metastasis of tumors through their ability to regulate the proliferation, differentiation, migration, and survival of these cancerous cells [22]. Huang et al. [23] found that increasing the expression of the downregulated lncRNA LINC00961 resulted in increased Bax expression and the corresponding apoptotic death of NSCLC cells. Xu et al. further provided evidence suggesting that the lncRNA HULC is able to promote LSCC cell proliferation owing to its ability to PTPRO-dependent phosphorylation and activation of NF-κB [24]. Similarly, Wang et al. found that increased expression of the lncRNA MIR31HG in NSCLC led to enhanced tumor cell gefitinib resistance owing to associated activation of the EGFR/PI3K/AKT signaling pathway [25]. Li J et al. reported lncRNA-ATB overexpression may promote the progression of LSCC by modulating the microRNA-590-5p/NF-90 axis [26]. To improve the prediction accuracy, we analyzed high-throughput data and were thereby able to identify two upregulated lncRNAs (LINC02555 and APCDD1L-DT) and one downregulated lncRNA (OTX2-AS1) in LSCC patients, all three of which were significantly associated with patient clinical outcomes.

Up to now, some researches have demonstrated that cigarette smoking or exposure may be associated with the expression of lncRNAs in lung cancer patients. Li J et al. [27] found that polymorphisms in lncRNA AC016683.6 significantly increased the risk of lung cancer in the smoking population. Lv X et al. also reported that there were significant interactions of lncRNA AC008392.1 polymorphisms with smoking exposure to lung cancer susceptibility [28]. Moreover, Chen Y et al. suggested that smoking-associated lncRNAs have a role in various processes and pathways, including cell proliferation and the cyclic guanosine monophosphate cGMP)/protein kinase cGMP-dependent 1 signaling pathway via bioinformatics analysis [29]. However, the association between cigarette smoking exposure and the identified lncRNAs (LINC02555, APCDD1L-DT and OTX2-AS1) in present study has not been investigated. Further studies are needed to confirm these predictions.

We further sought to gain insight into the functional importance of the three lncRNAs identified in this study via using a co-expression analysis-based approach to identify putative lncRNA target genes that were then subjected to GO and KEGG enrichment analyses. This approach revealed the lncRNA-associated target genes to be enriched for functionality in the context of endocytosis, focal adhesion, MAPK signaling, and lysosomal activity, all of which are closely linked with oncogenesis and tumor progression [30, 31]. To date no studies have specifically studied LINC02555, APCDD1L-DT, or OTX2-AS1 in the context of LSCC. As such, future in-depth molecular analyses will be needed to confirm the findings of our co-expression analysis.

There are multiple limitations to the present study. For one, these results are derived solely from bioinformatics analyses and as such necessitate additional functional validation. Furthermore, we did not explore the molecular mechanisms linking the expression of these three lncRNAs to LSCC patient prognosis, and as such future experimental studies will be required in order to elucidate these mechanisms. As such, large-scale multi-center trials will be essential in order to validate and expand upon our findings.

## Conclusions

In summary, in the present article we were able to identify three different lncRNAs that could be used to predict survival outcomes in patients with LSCC. Further large-scale multi-center trials will be needed to confirm our findings, and to explore the molecular mechanisms linking these lncRNAs to clinical outcomes in LSCC patients. While much work is still required before this lncRNA signature can be implemented in a clinical setting, we nonetheless feel that our findings may have significant value as a future diagnostic or prognostic tool in the context of LSCC patient identification and care.

## Supplementary Information

**Additional file 1 Fig S1.** Three lncRNAs based risk score distribution, patients’ event-free survival time and a heatmap of the expression profiles of the three lncRNA. (a) Red dots represent the high-risk group and green dots represent the low-risk group (b) Red dots represent the dead group and green dots represent the alive group: a population-based study with a mean follow-up of 3.5 years.

## Abbreviations

LSCC: Lung squamous cell carcinoma
LncRNAs: Long non-coding RNAs
TCGA: The Cancer Genome Atlas
PCGs: Protein-coding genes
ROC: Receiver operating characteristic
OS: Overall survival
FCs: Fold changes
KEGG: Kyoto Encyclopedia of Genes and Genomes
GO: Gene Ontology
BP: Biological process
NF-κB: Nuclear factor-κB
