# Supplementary material for: Identification of a prognostic long noncoding RNA signature in lung squamous cell carcinoma: a population-based study with a mean follow-up of 3.5 years
Source: Arch Public Health. 2021 Apr 28;79:61. doi: 10.1186/s13690-021-00588-2 (PMC8082628; doi:10.1186/s13690-021-00588-2)
Supplement: Supplementary file 1 — Additional file 1 Fig S1. Three lncRNAs based risk score distribution, patients’ event-free survival time and a heatmap of the expression profiles of the three lncRNA. (a) Red dots represent the high-risk group and green dots represent the low-risk group (b) Red dots represent the dead group and green dots represent the alive group: a population-based study with a mean follow-up of 3.5 years. [file 13690_2021_588_MOESM1_ESM.docx]

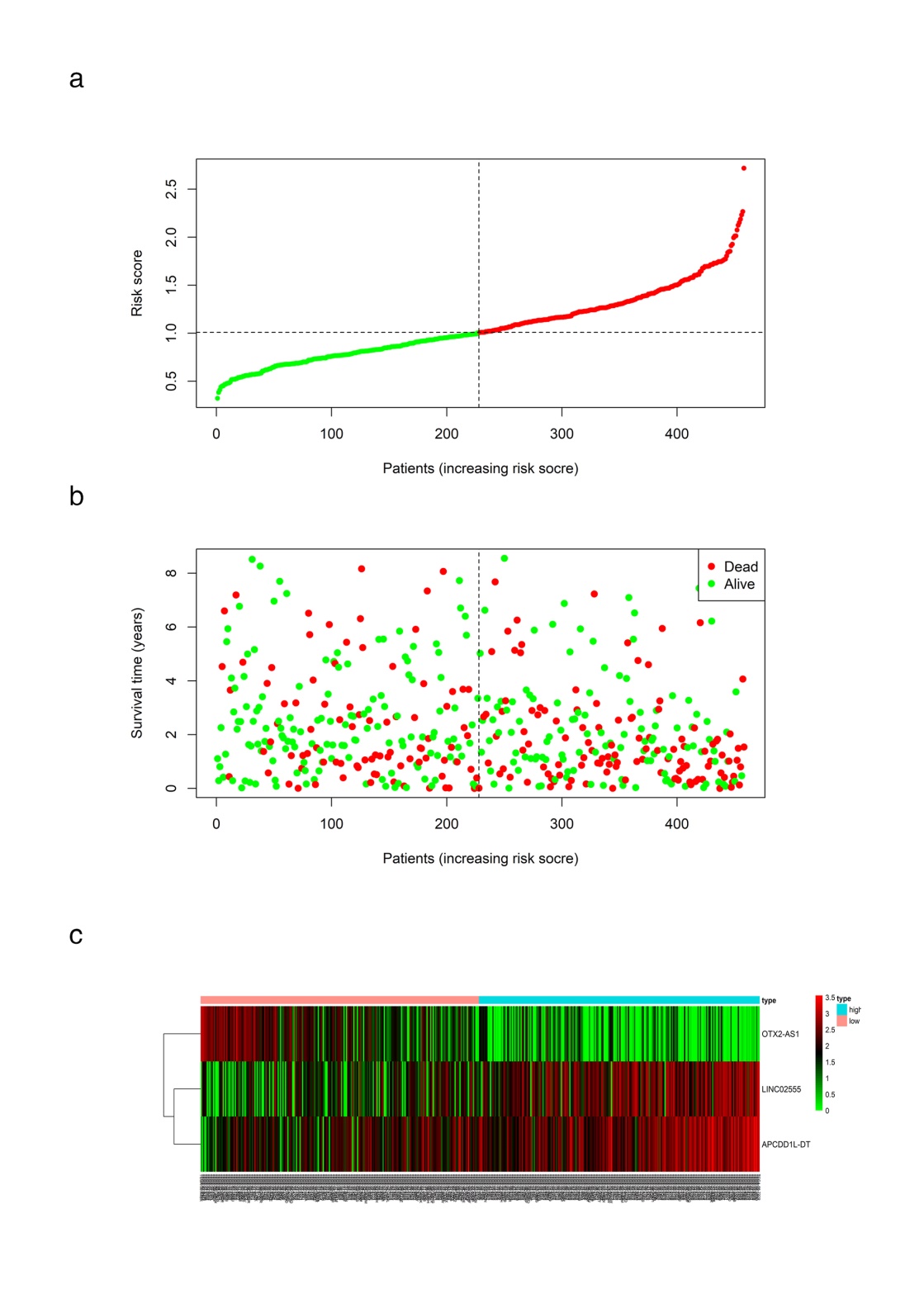


Fig S1. Three lncRNAs based risk score distribution, patients’ event-free survival time and a heatmap of the expression profiles of the three lncRNA. (a) Red dots represent the high-risk group and green dots represent the low-risk group (b) Red dots represent the dead group and green dots represent the alive group: a population-based study with a mean follow-up of 3.5 years
